# CAR‐T therapy as a consolidation in remission B‐ALL patients with poor prognosis

**DOI:** 10.1002/cnr2.1706

**Published:** 2022-08-22

**Authors:** Zhichao Yin, Yuehui Lin, Dan Liu, Chunrong Tong, Shuangyou Liu

**Affiliations:** ^1^ Department of Hematology Beijing Boren Hospital Beijing China

**Keywords:** B‐cell acute lymphoblastic leukemia, CAR T‐cells, complete remission

## Abstract

**Background:**

To date, almost all studies regarding chimeric antigen receptor (CAR)‐T cell therapy for B‐cell acute lymphoblastic leukemia (B‐ALL) were performed in refractory/relapsed (r/r) or minimal residual disease‐positive patients. CAR‐T therapy in remission patients has not been reported.

**Aim:**

To observe the treatment outcome of CAR‐T cells for remission B‐ALL patients with poor prognosis.

**Methods and Results:**

CAR‐T treatment was applied to two B‐ALL patients in remission status who had poor prognostic factors and refused transplantation, and one case was unable to accept standard chemotherapy owing to multiple complications. The procedure of CAR‐T therapy in these two remission patients was the same as that in r/r B‐ALL patients. Lentiviral vectors encoding second generation CARs composed of CD3ζ and 4‐1BB were used to produce CAR‐T cells. Lymphodepleting agents fludarabine and cyclophosphamide were administered prior to cell infusion. Grade I cytokine release syndrome occurred after each T‐cell infusion and there was no neurotoxicity. CAR‐T treatment followed by non‐intensive maintenance chemotherapy and targeted drugs allowed both patients to obtain a long‐term event‐free survival of more than three and a half years without transplantation.

**Conclusions:**

CAR‐T therapy could be used in high‐risk B‐ALL patients as a consolidation to avoid transplantation, the combination of CAR‐T and following maintenance therapy may be better than CAR‐T alone for durable remission.

## 1 INTRODUCTION

In recent years, chimeric antigen receptor (CAR) T‐cell therapy targeting CD19 or CD22 antigen has been used as a potent approach to treat refractory/relapsed (r/r) B‐cell acute lymphoblastic leukemia (B‐ALL) and achieved remarkable efficacy. The high complete remission (CR) rates were obtained in both pediatric and adult r/r B‐ALL patients treated by either CD19‐directed (81%–90%) or CD22‐directed (73%–80%) CAR‐T cells.^1^, ^2^, ^3^, ^4^, ^5^, ^6^ With two CD19 CAR‐T cell products being approved by U.S. Food and Drug Administration for clinical use in 2017, CD19 CAR‐T therapy has been administered in more and more medical centers for treating r/r B‐cell malignancies.

However, to date, all published studies regarding CAR‐T therapy for B‐ALL were performed in refractory/relapsed or minimal residual disease (MRD)‐positive patients. Here, CAR‐T treatment was applied to two B‐ALL patients in complete remission status as a consolidation for preventing relapse. Both cases had adverse prognostic factors^7^ and refused transplantation, one of them could not receive standard chemo regimens owing to multiple complications. CAR‐T treatment was implemented in the first year of diagnosis, then followed by non‐intensive maintenance chemotherapy and targeted drugs. As of the last follow‐up, both patients remained in MRD negative remission and survived for more than three and a half years without transplantation.

This pilot observation was approved by Beijing Boren Hospital ethics committee and informed consents were obtained in accordance with the Declaration of Helsinki.

## 2 PATIENTS AND TREATMENTS

**FIGURE 1:**
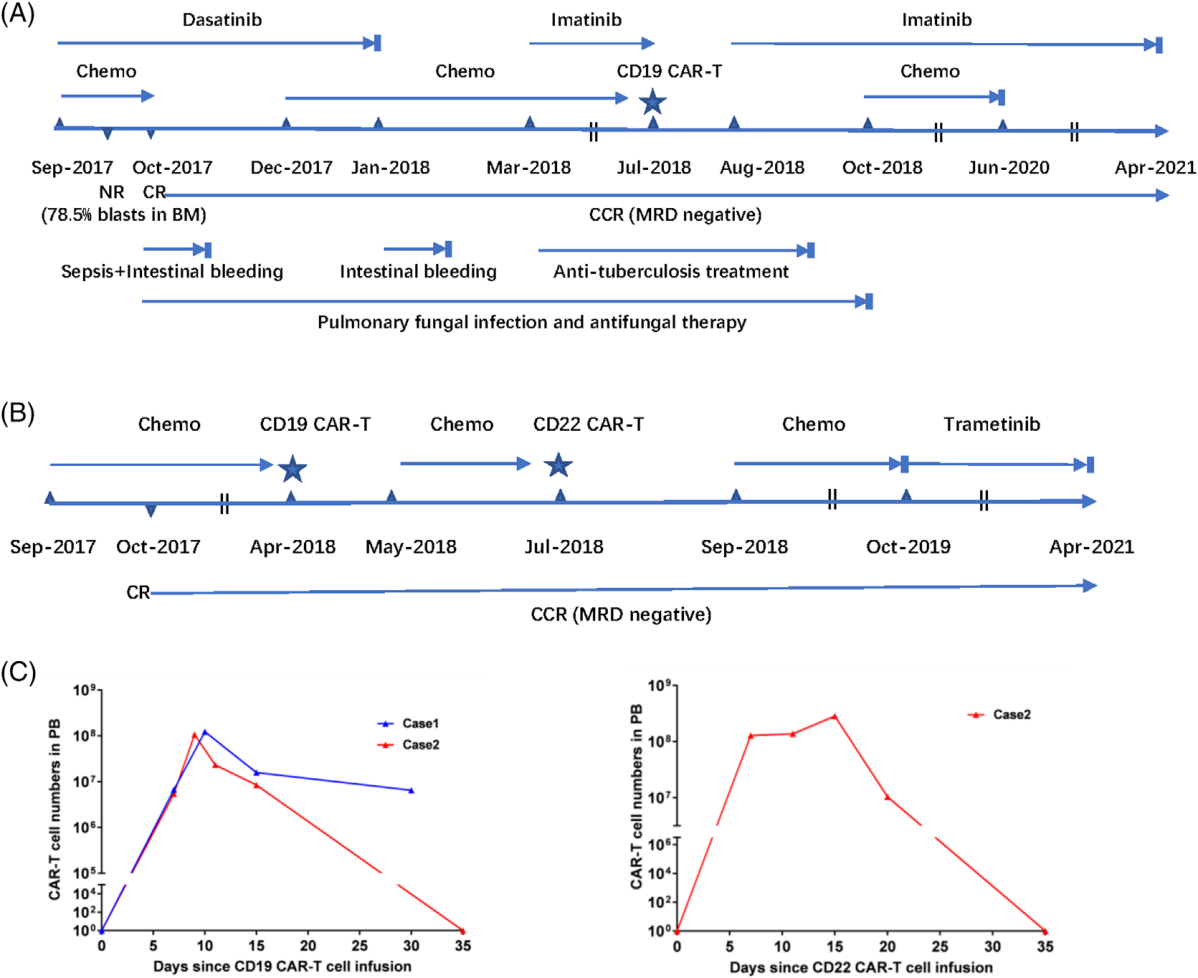
Treatment summary and CAR‐T cell expansion in peripheral blood (PB). (A) Treatment summary of case 1. On day 15 after induction chemotherapy and dasatinib, the patient showed no response (NR) with 78.5% lymphoblasts in bone marrow (BM). On day 34, complete remission (CR) was achieved. CD19 CAR‐T cells were infused in the first year from diagnosis. Anti‐tuberculosis treatment lasted for 6 months, and antifungal therapy lasted for 1 year. Chemotherapy was stopped after 2 years and 8 months of CR. Imatinib was stopped after three and a half years of CR. (B) Treatment summary of case 2. CD19 and CD22 CAR‐T cells were sequentially infused in the first year from diagnosis. Chemotherapy was stopped after 2 years of CR, then low‐dose trametinib (0.5–1 mg/day) alone was administered for another one and a half years. (C) CAR‐T cell expansion in PB. CAR‐T cells were assayed by flow cytometry. After each infusion, cell proliferation was observed with a high peak level of over 1.0 × 10^8^/L, and CAR‐T cells were undetectable between 35 and 72 days. CCR, continuous complete remission; MRD, minimal residual disease

Patient 1 is a boy. He was diagnosed with B‐ALL at the age of 5 years in September 2017, presenting with a high white blood cell count of 152.26 x 10^9^/L, the chromosomal translocation t (9;22) and BCR/ABL1 fusion gene (p190). The induction chemotherapy and dasatinib were immediately administered. At day 15 evaluation, his bone marrow (BM) still showed 78.5% lymphoblasts. On day 34, the patient achieved MRD‐negative remission. However, at the end of induction, he suffered from sepsis (enterobacter aerogenes), pulmonary infection (Figure S1, there were multiple round nodules in the lungs and histoplasma infection was considered) and lower intestinal bleeding. The standard chemotherapy could not be followed as planned, then dasatinib alone was taken for 2 months. When his sepsis and intestinal hemorrhage resolved while pulmonary fungal infection remained unsolved, non‐intensive chemodrugs were administered, including regimens VD (vindesine and dexamethasone), MTX (methotrexate 2‐3 g/m^2^), oral MTX plus 6‐mercaptopurine (6‐MP), LAM (l‐asparaginase, low‐dose cytarabine and 6‐MP) and VLD (vindesine, l‐asparaginase and dexamethasone). Unfortunately, lower intestinal bleeding occurred again after 1 month of chemo. Colonoscopy showed colitis and biopsy revealed chronic colitis with focal lymphadenosis and granulomatosis, tuberculosis was suspected (Figure S2). Considering that the bleeding might be related to dasatinib as well (no direct evidence of tuberculosis was found), dasatinib was stopped for 2 months and thereafter replaced by imatinib (Figure 1A). The anti‐tuberculosis treatment lasted for 6 months (isoniazid, pyrazinamide and ethambutol, 2 months; isoniazid and pyrazinamide, 2 months; isoniazid only, 2 months), rifampicin was not administered to this patient for avoiding its influence on tyrosine kinase inhibitors. The antifungal therapy lasted for 1 year.

Since this boy had high‐risk factors and had not received standard chemotherapy due to multiple complications, we planned to add CAR‐T therapy for preventing relapse after discussed with his parents who refused transplantation. On July 17, 2018, when his pulmonary fungal infection obviously improved, CD19 CAR‐T cells were infused at a dose of 3 x 10^5^/kg. The process was going well, there was grade I cytokine release syndrome (CRS) and no neurotoxicity. One month later, imatinib was taken again. Three months later, he continued to receive the maintenance chemotherapy, including non‐intensive consolidation (once every 3–4 months and each lasting for 1–2 weeks) and oral chemodrugs at home (6‐MP/day, MTX/week and dexamethasone 5 days/month). Chemotherapy was stopped in June 2020 after 2 years and 8 months of CR. Then, he was given imatinib alone until April 2021 after three and a half years since CR (Figure 1A).

Patient 2 is a girl. She was diagnosed with B‐ALL at age of 18 in September 2017, with 4 Ph‐like gene mutations in IKZF1, K‐RAS and N‐RAS (Table S1). She obtained CR after induction chemotherapy and continued to receive chemodrugs. The Ph‐like B‐ALL has been verified as a high‐risk subtype with a poor survival in both adults and children,^8^, ^9^ chemotherapy alone may not achieve a long‐term remission in this girl harboring Ph‐like gene mutations. Then, hematopoietic cell transplantation was recommended but refused by the girl and her family. Alternative CAR‐T immunotherapy might improve the prognosis and they all agreed to receive CAR‐T cells. Subsequently, CD19 CAR‐T treatment was administered with a dose of 3 x 10^5^/kg on April 9, 2018. Considering that the girl aged over 18 years old and had 4 kinds of Ph‐like gene mutations, CD22 CAR‐T cells were followed 3 months later at a dose of 5 x 10^5^/kg. Grade I CRS occurred in both infusions and there was no neurotoxicity. After CAR‐T therapy, she continued to receive non‐intensive chemodrugs like case 1. Chemotherapy was stopped in October 2019 after 2 years of CR, then low‐dose trametinib (0.5‐1 mg/day, when she had moderate rash, trametinib was reduced to 0.5 mg/day) was taken for another one and a half years. As of the last follow‐up in April 2021, the girl had been in continuous MRD‐negative CR for 3 years and a half (Figure 1B).

## 3 CAR‐T THERAPY

The procedure of CAR‐T therapy in these two CR patients was the same as that in r/r B‐ALL patients. Murine CD19 and humanized CD22 CARs were lentiviral vectors encoding second generation CAR composed of CD3ζ and 4‐1BB, provided by Shanghai YaKe Biotechnology Ltd. (Shanghai, China).^10^ Patient‐derived cells were collected for producing CAR‐T cells, which were transfected and cultured for 5–8 days in the cytology laboratory of Beijing Boren Hospital (Beijing, China). Lymphodepleting agents fludarabine (30 mg/m^2^/day) and cyclophosphamide (250 mg/m^2^/day) were given for 3 days prior to each cell infusion. CAR‐T cells (FITC‐CAR+ PerCP‐CD3+ T cells, Figure S3) were assayed through flow cytometric quantitation (FITC‐conjugated CD19‐ or CD22‐CAR detection reagents were also from Shanghai YaKe Biotechnology Ltd.). MRD was detected by flow cytometry (FCM) (BD FACSCanto II) and quantitative PCR for BCR/ABL1 fusion gene (Applied Biosystems 7500, Thermo Fisher Scientific), the MRD level below 1 x 10^−4^ (both FCM and qPCR) was identified as negativity. B‐cell aplasia (BCA) was defined as less than 3% CD19/CD22 positive lymphocytes in BM. CRS was graded through Penn grading scale.^11^

After each infusion, CAR‐T cell expansion in peripheral blood (PB) was observed with a high peak level of over 1.0 × 10^8^/L, and CAR‐T cells were also present in BM and cerebrospinal fluid (Figure 1C and Table 1). Although CAR‐T cells in PB could not be detected by FCM (no qPCR data) in 35–72 days, B‐cell aplasia, a surrogate marker of functional CAR‐T cells, had persisted for 21 (case 2) and 27 (case 1) months since CD19 CAR‐T cell infusion.

**TABLE 1:** CAR‐T therapy

Variables	Case 1	Case 2	
	CD19 CAR‐T	CD19 CAR‐T	CD22 CAR‐T
Rationale of CAR‐T therapy	WBC 152.26 x 10^9^/L at diagnosis; t (9;22); 78.5% lymphoblasts in BM at day 15 after chemo and dasatinib; pulmonary fungal infection; intestinal bleeding with suspected tuberculosis infection	4 Ph‐like gene mutations in IKZF1, K‐RAS and N‐RAS^a^	
Date of T‐cell infusion	July 17, 2018	April 9, 2018	July 10, 2018
Dose of T‐cell infusion	3 x 10^5^/kg	3 x 10^5^/kg	5 x 10^5^/kg
Cytokine release syndrome	1	1	1
Neurotoxicity	0	0	0
Peak CAR‐T cell numbers in PB	1.23 × 10^8^/L(D10)	1.07 × 10^8^/L(D9)	2.84 × 10^8^/L(D15)
Detectable CAR‐T cells in BM/CSF between day 15–35	Yes/yes	Yes/yes	Yes/yes
Duration of B‐cell aplasia after CD19 CAR‐T	27 months	21 months	

Abbreviations: BM, bone marrow; CSF, cerebrospinal fluid; PB, peripheral blood; WBC, white blood cell.

^a^ Details in Table S1.

## 4 DISCUSSION

So far, CAR‐T cell therapy has usually been used to treat refractory/relapsed B‐ALL. Currently, a clinical trial putting CD19 CAR‐T cells in high‐risk B‐ALL patients with MRD positivity at the end of consolidation chemotherapy (an earlier intervention) is ongoing.^12^ The rationale of that we administered CAR‐T therapy in these two remission patients was that they had adverse prognostic factors (Table 1) and refused transplantation. For case 1, except for high‐risk factors, he could not tolerate the standard chemotherapy due to the infections and repeated intestinal bleeding after induction chemotherapy. Since children usually have a better prognosis than adults, this boy was given CD19 CAR‐T only. Case 2 is an adult and had four kinds of Ph‐like gene mutations, she was sequentially infused with CD19 and CD 22 CAR‐T cells.

Although CAR‐T cell therapy has achieved high complete remission rates in r/r B‐ALL, it could not maintain a durable remission in most cases and disease relapse remained a major problem in post CAR‐T patients.^1^, ^2^, ^3^, ^4^, ^5^ Therefore, maintenance chemodrugs were continuously used following CAR‐T in these two cases to avoid possible relapse. Under the circumstance of chemo use, the long duration of BCA in both patients was an unexpected result (their B‐cell recovery appeared after the termination of chemotherapy), indicating that non‐intensive chemodrugs seemed not to influence the function of CAR‐T cells. We and others have verified that steroids do not impact on the antitumor potency of CAR‐T cells.^13^, ^14^ It is surprising that, from these two cases, we observed that the non‐intensive maintenance chemotherapy did not affect the surviving of CAR‐T cells either.

In conclusion, we utilized CAR‐T treatment as a consolidation to two remission B‐ALL patients with poor prognosis, CAR‐T therapy followed by non‐intensive chemo and targeted drugs allowed them to obtain a long‐term event‐free survival of more than three and a half years without transplantation. Both patients had terminated all medications and gone back to normal life. This pilot observation provides some new insights into the use of CAR‐T therapy: it could be moved forwards in high‐risk patients to avoid relapse and transplantation (importantly, CAR‐T therapy is much safe in CR patients); the combination of CAR‐T and following maintenance therapy may be better than CAR‐T alone without further treatments for durable remission. More cases need to be further investigated to verify these phenomena.

## AUTHOR CONTRIBUTIONS

**Zhichao Yin:** Conceptualization (equal); data curation (equal); resources (equal); writing – original draft (equal). **Yuehui Lin:** Conceptualization (equal); data curation (equal); resources (equal). **Dan Liu:** Investigation (equal); resources (equal). **Chunrong Tong:** Conceptualization (equal); supervision (equal). **Shuangyou Liu:** Conceptualization (equal); data curation (equal); supervision (equal); writing – review and editing (lead).

## CONFLICT OF INTEREST

The authors declare no conflict of interest.

## ETHICS STATEMENT

This study was approved by Beijing Boren Hospital ethics committee.

## PATIENT CONSENT STATEMENT

Informed consents were obtained in accordance with the Declaration of Helsinki.

## Supporting information


**FIGURE S1** Chest CT images of case 1
**FIGURE S2** Colonoscopy and colon biopsy of case 1
**FIGURE S3** Representative flow cytometry plots of CAR‐T cells
**TABLE S1** Gene mutations in case 2
**TABLE S2** Complete blood count before and after CAR‐T therapy

## Data Availability

For original data, please contact Zhichao Yin by e‐mail at yinzc@borenhospital.com or corresponding author.
