# Supplementary material for: CAR‐T therapy as a consolidation in remission B‐ALL patients with poor prognosis
Source: Cancer Rep (Hoboken). 2022 Aug 22;5(10):e1706. doi: 10.1002/cnr2.1706 (PMC9575491; doi:10.1002/cnr2.1706)
Supplement: Supplementary file 1 — FIGURE S1 Chest CT images of case 1 FIGURE S2 Colonoscopy and colon biopsy of case 1 FIGURE S3 Representative flow cytometry plots of CAR‐T cells TABLE S1 Gene mutations in case 2 TABLE S2 Complete blood count before and after CAR‐T therapy [file CNR2-5-e1706-s001.pdf]

Supporting Information:

Supplemental figure legends

Figure S1 Chest CT images of case 1

Figure S2 Colonoscopy and colon biopsy of case 1

Figure S3 Representative flow cytometry plots of CAR-T cells

Table S1 Gene mutations in case 2

Table S2 Complete blood count before and after CAR-T therapy

Supplemental figure legends

Figure S1. Chest CT images of case 1. (A) There were multiple round nodules in the lungs and histoplasma infection was considered. These images were taken in December, 2017, two months after pulmonary infection was diagnosed (initial CT images were lost due to computer virus). After one year of antifungal treatment, the boy's chest CT scans gradually improved (B) and eventually returned to normal (C).

Figure S2. Colonoscopy and colon biopsy of case 1. Colonoscopy showed colitis with edema, multiple erythema, erosion, mucosal friability bleeding in the transverse colon, splenic flexure, descending colon, sigmoid and rectum, the severe lesions were located in the sigmoid (A) and descending colon (B). Colon biopsy revealed chronic colitis with focal lymphadenosis and granulomatosis, showed in (C) H&E stain 10X and (D) H&E stain 40X, tuberculosis was suspected.

Figure S3. Representative flow cytometry plots of CAR-T cells. Both FITC-CAR+ and PerCP-CD3+ cells are CAR-T cells.

Figure S1

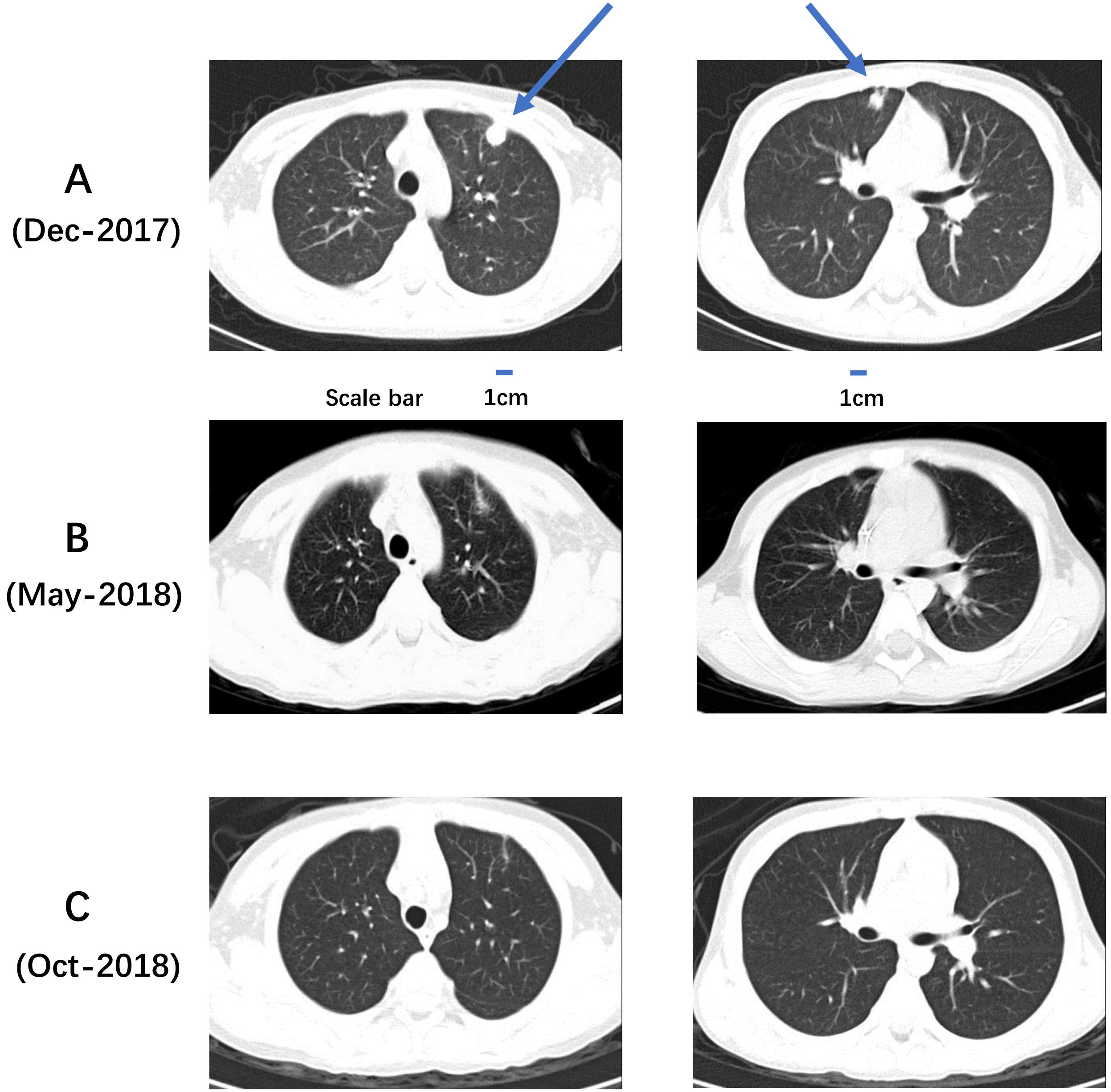

Figure S2

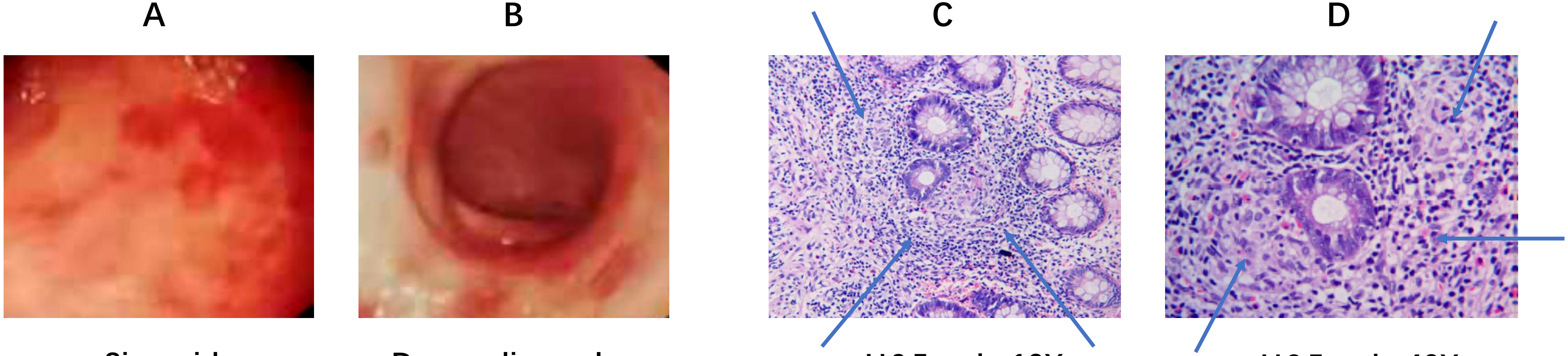

A — Sigmoid

B — Descending colon

C — H&E stain 10X

D — H&E stain 40X

Figure S3

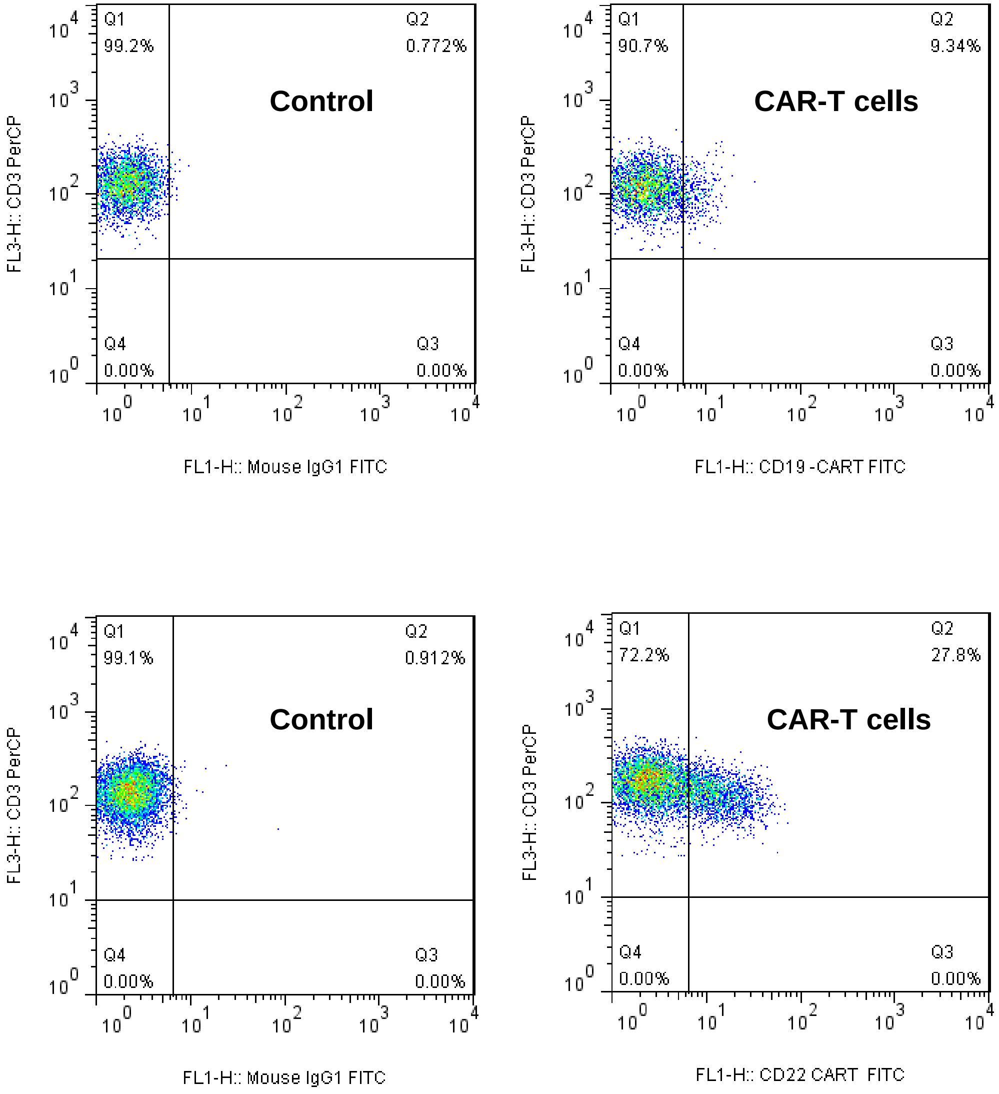

Q1
99.2%
Q2
0.772%
Control
Q4
0.00%
Q3
0.00%
FL3-H:: CD3 PerCP
FL1-H:: Mouse IgG1 FITC
Q1
90.7%
Q2
9.34%
CAR-T cells
Q4
0.00%
Q3
0.00%
FL3-H:: CD3 PerCP
FL1-H:: CD19 -CART FITC
Q1
99.1%
Q2
0.912%
Control
Q4
0.00%
Q3
0.00%
FL3-H:: CD3 PerCP
FL1-H:: Mouse IgG1 FITC
Q1
72.2%
Q2
27.8%
CAR-T cells
Q4
0.00%
Q3
0.00%
FL3-H:: CD3 PerCP
FL1-H:: CD22 CART FITC

Table S1. Gene* mutations in case 2

No.	Mutation location	Exon	Frequency of mutation
1	NM_006060(IKZF1): c.1387dupA(p.Y462fs)	8	1.63%
2	NM_004985(KRAS): c.34G>A(p.G12S)	2	8.26%
3	NM_002524(NRAS): c.37G>T(p.G13C)	2	3.43%
4	NM_002524(NRAS): c.34G>A(p.G12S)	2	9.92%

*127 genes related to hematological neoplasm were screened by next generation sequencing (NGS) with the sample at diagnosis.

Table S2. Complete blood count before and after CAR-T therapy

Variables	Case 1		Case 2			
	Before CD19 CAR-T	After CD19 CAR-T	Before CD19 CAR-T	After CD19 CAR-T	Before CD22 CAR-T	After CD22 CAR-T
White blood cell count （$x10^9$/L）	2.72	2.44	3.1	2.08	2.68	2.56
Neutrophil count （$x10^9$/L）	1.72	1.48	1.15	1.18	1.39	1.03
Neu%	63.24	60.66	37.10	56.73	51.87	40.23
Lymphocyte count （$x10^9$/L）	0.68	0.39	1.39	0.44	0.86	1.05
Lym%	25.00	15.98	44.84	21.15	32.09	41.02
CD3+ T cell/ Lymphocyte (%)	83.8	82.4	N/A	75.2	N/A	87.7
CD19/22+ B cell /Lymphocyte (%)	1.88	0	N/A	0	N/A	0
Haemoglobin (g/L)	97	102	110	116	95	98
Platelet （$x10^9$/L）	284	227	229	166	231	48

N/A: not applicable (no data). In case 2, after CD22 CAR-T, there were no CD19 or CD22 positive cells.
